# Supplementary material for: Kin discrimination in social yeast is mediated by cell surface receptors of the Flo11 adhesin family
Source: eLife. 2020 Apr 14;9:e55587. doi: 10.7554/eLife.55587 (PMC7156268; doi:10.7554/eLife.55587)
Supplement: Supplementary file 1. [file elife-55587-supp1.docx]

**Supplementary File 1**

**Flo11-type A domain sequences used for phylogenetic analysis.**

| **Strain^1^** | **Accession number^2,3^** | **Protein sequence** | **Source or Reference** |
| --- | --- | --- | --- |
| S288c | P08640^2^ | GCPNLDFNWHMDQQNIMQYTLDVTSVSWVQDNTYQITIHVKGKENIDLKYLWSLKIIGVTGPKGTVQLYGYNENTYLIDNPTDFTATFEVYATQDVNSCQVWMPNFQIQFEYLQGSAAQYASSWQWGTTSFDLSTGCNNYDNQGHSQTDFP | UniProtKB |
| Σ1278b | E9P9G2^2^ | GCPNLDFNWHMNQQNIMQYTLDVTSVSWVQDNTYQITIHVKGEENIDLKYLSSLKIIGVTGPKDTVQLYGYDENTDWIDNPLVSRCDENTYLIDNPTDFTATFEVYATQDVNSCQVWMPNFQIQFEYLQGSAAEYACSWEWGTTSFYLSTGCDNYDNQGYSQTDFP | UniProtKB |
| YJM789 | A6ZVT8^2^ | GCPNLDFNWHMNQQNIMQYTLDVTSVSWVQDNTYQITIHVKGEENIDLEYLSSLKIIGVTGPKDTVQLYGYNEDTYLIDNPLVSRCDEYTYLIDNPTDFTATFEVYATQDVNSCQVWMPNFQIQFEYLQDIAPEDECSWEWGTTSFTLSTGCDNYDNQGYSQTDFP | UniProtKB |
| K7 | 2WFU7^2^ | GCPNLDFNWHMNQQNIMQYTLDVTSVSWVQDNTYQITIHVKGEENIDLKYLWSLKIIGVTGPKDTVQLYGYDEDTDWIDNPLVSRCDENTHLIDNPTDFTATFEVYATQDVNSCQVWMPNFQIQFEYLQGSAAEHACSWEWGTTSFYLSTGCDNYDNQGYSQTDFP | UniProtKB |
| RM11-1a | B3LTJ0^2^ | GCPNLDFNWHMNQQTIMQYTLDVTSVSWVQDNTYQITIHVKGKENIDLNYLSSLKIIGVTGPKDTVQLYGCNENTYLIDNPTDFTATFEVYATQDVNSCQVWMPNFQIQFEYLQGSAAQYASSWKWGTTSFNLSTGCNNYDNQGHSQTDFP | UniProtKB |
| 133d | E9P8M0^2^ | GCPNLDFNWHMNQQTIMQYTLDVTSVSWVQDNTYQITIHVKGKENIDLKYLRSLKIIGVTGPKGTVQLYGYNENTYLIDNPTDFTATFEVYATQDVNSCQVWMPNFQIQFEYLQGSAAQYASSWKWGTTSFYLSTGCNNYDNQGHSQTDFP | UniProtKB |
| Lalvin_EC1118 | C8ZAR8^2^ | GCPNLDFNWHMNQQTIMQYTLDVTSVSWVQDNTYQITIHVKGKENIDLNYLSSLKIIGVTGPKDTVQLYGCNENTYLIDNPTDFTATFEVYATQDVNSCQVWMPNFQIQFEYLQGSAAQYASSWKWGTTSFNLSTGCNNYDNQGHSQTDFP | UniProtKB |
| FostersO | E7NPK4^2^ | GCPNLDFNWHMNQQNIMQYTLDVTSVSWVQDNTYQITIHVKGEENIDLKYLSSLKIIGVTGPKDTVQLYGYDENTDWIXNPLVSRCDENTYLIDNPTDFTATFEVYATQDVNSCQVWMPNFQIQFEYLQGSAAEYACSWEWGTTSFYLSTGCDNYDNQGYSQTDFP | UniProtKB |
| AWRI796 | B5VKV5^2^ | GCPNLDFNWHMNQQTIMQYTLDVTSVSWVQDNTYQITIHVKGKENIDLKYLWSLKIIGVTGPKGTVQLYGYNENTYLIDNPTDFTATFEVYATQDVNSCQVWMPNFQIQFEYLQGSAAQYASSWKWGTTSFDLSTGCNNYDNQGHSQTDFP | UniProtKB |
| P283 | W7RDJ2^2^ | GCPNLDFNWHMNQQTIMQYTLDVTSVSWVQDNTYQITIHVKGKENIDLNYLSSLKIIGVTGPKDTVQLYGCNENTYLIDNPTDFTATFEVYATQDVNSCQVWMPNFQIQFEYLQGSAAQYASSWKWGTTSFNLSTGCNNYDNQGHSQTDFP | UniProtKB |
| SK1 |  | GCPNLDFNWHMDQQNIMEYTLDVTSVSWVQDNTYQITVHVKGKENIDLKYLWSLKIIGVTGPKGTVQLYGYNENTYLIDNPTDFTATFEVYATQDVNSCQVWMPNFQIQFEYLQGSAAQYASSWKWGTTSFDLSTRCNNYDNQGHSQTDFP | SGRP (Liti et al., 2009),  Sanger Institute |
| W303 |  | GCPNLDFNWHMDQQNIMQYTLDVTSVSWVQDNTYQITIHVKGKENIDLKYLWSLKIIGVTGPKGTVQLYGYNENTYLIDNPTDFTATFEVYATQDVNSCQVWMPNFQIQFEYLQGSAAQYASSWQWGTTSFDLSTGCNNYDNQGHSQTDFP | SGRP (Liti et al., 2009),  Sanger Institute |
| 322134S |  | GCPNLDFNWHMNQQNIMQYTLDVTSVSWVQDNTYQITIHVKGKENIDLKYLSSLKIIGVTGPKDTVQLYGYNENTYLIDNPLVSRCDENTYLIDNPTDFTATFEVYATQDVN | SGRP (Liti et al., 2009),  Sanger Institute |
| YS2 |  | GCPNLDFNWHMNQQNIMQYTLDVTSVSWVQDNTYQITIHVKGEENIDLKYLSSLKIIGVTGPKDTVQLYGYDENTDWIDNPLVSRCDENTYLIDNPTDFTATFEVYATQDVNS | SGRP (Liti et al., 2009),  Sanger Institute |
| YS9 |  | GCPNLDFNWHMNQQNIMQYTLDVTSVSWVQDNTYQITIHVKGEENIDLKYLSSLKIIGVTGPKDTVQLYGYDENTDWIDNPLVSRCDENTYLIDNPTDFTATFEVYATQDVNS | SGRP (Liti et al., 2009),  Sanger Institute |
| BC187 |  | GCPNLDFNWHMNQQTIMQYTLDVTSVSWVQDNTYQITIHVKGKENIDLNYLSSLKIIGVTGPKDTVQLYGYNENTYLIDNPTDFTATFEVYATQDVNSC | SGRP (Liti et al., 2009),  Sanger Institute |
| DBVPG1106 |  | GCPNLDFNWHMNQQTIMQYTLDVTSVSWVQDNTYQITIHVKGKENIDLKYLWSLKIIGVTGPKGTVQLYGYNENTYLIDNPTDFTATFEVYATQDVNSC | SGRP (Liti et al., 2009),  Sanger Institute |
| DBVPG6765 |  | GCPNLDFNWHMNQQTIMQYTLDVTSVSWVQDNTYQITIHVKGKENIDLNYLWSLKIIGVTGPKGTVQLHSYNENTYLIDNPTDFTATFEVYATQDVNSCQVWMPDFQIQFEYLQDSAAQYASSWKWGTTSFNLSTGCNNYDNQGHSQTDFP | SGRP (Liti et al., 2009),  Sanger Institute |
| DBVPG6040 |  | GCPNLDFNWHMNQQTIMQYTLDVTSVSWVQDNTYQITIHVKGKENIDLNYLSSLKIIGVTGPKDTVQLYGCNENTYLIDNPTDFTATFEVYATPDVNSCQVWMPNFQIQFEYLQDSAAQYASSWKWGTTSFALSTGCNNYDDQGYSQTDFP | SGRP (Liti et al., 2009),  Sanger Institute |
| DBVPG1788 |  | GCPNLDFNWHMNQQTIMQYTLDVTSVSWVQDNTYQITIHVKGKENIDLKYLWSLKIIGVTGPKGTVQLYGYNENTYLIDNPTDFTATFEVYATQDVNSCQVWMPNFQIQFEYLQGSAAQYASSWKWGTTSFDLSTGCNNYDNQGHSQTDFP | SGRP (Liti et al., 2009),  Sanger Institute |
| UWOPS03_461.4 |  | GCPNLDFNWHMNQQNIMQYTLDVTSVSWVQDNTYQITIHVKGKENIDLKYLWSLKIIGVTGPKGTVQLYGYNENTYLIDNPTDFTATFEVYATQDVNSCQVWMPNFQIQFEYLQGSAAQYASSWKWGTTSFDLSTGCNNYDNQGHSQTDFP | SGRP (Liti et al., 2009),  Sanger Institute |
| UWOPS05_217.3 |  | GCPNLDFNWHMNQQNIMQYTLDVTSVSWVQDNTYQITIHVKGKENIDLKYLWSLKIIGVTGPKGTVQLYGYNENTYLIDNPTDFTATFEVYATQDVNSCQVWMPNFQIQFEYLQGSAAQYASSWKWGTTSFDLSTGCNNYDNQGHSQTDFP | SGRP (Liti et al., 2009),  Sanger Institute |
| UWOPS05_227.2 |  | GCPNLDFNWHMNQQNIMQYTLDVTSVSWVQDNTYQITIHVKGKENIDLKYLWSLKIIGVTGPKGTVQLYGYNENTYLIDNPTDFTATFEVYATQDVNSCQVWMPNFQIQFEYLQGSAAQYASTWKWGTTSFDLSTGCNNYDNQGHSQTDFP | SGRP (Liti et al., 2009),  Sanger Institute |
| K11 |  | GCPNLDFNWHMNQQNIMQYTLDVTSVSWVQDNTYQITIHVKGEENIDLKYLSSLKIIGVTGPKDTVQLYGYHENTYLIDNPIDFVRCSENTHLIDNPTDFTATFEVYATQDVN | SGRP (Liti et al., 2009),  Sanger Institute |
| Y9 |  | GCPNLDFNWHMNQQNIMQYTLDVTSVSWVQDNTYQITIHVKGEENIDLKYLWSLKIIGVTGPKDTVQLYGYDEDTDWIDNPLVSRCDENTHLIDNPTDFTATFEVYA | SGRP (Liti et al., 2009),  Sanger Institute |
| Y12 |  | GCPNLDFNWHMNQQNIMQYTLDVTSVSWVQDNTYQITIHVKGEENIDLKYLWSLKIIGVTGPKDTVQLYGYDEDTDWIDNPLVSRCDENTHLIDNPTDFTATFEVYATQDVNS | SGRP (Liti et al., 2009),  Sanger Institute |
| YPS606 |  | GCPNLDFNWHMDQQNIMQYTLDVTSVSWVQDNTYQITIHVKGKENIDLKYLWSLKIIGVTGPKGTVQLYGYNENTYLIDNPTDFTATFEVYATQDVNSCQVWMPNFQIQFEYLQGSAAQYASSWKWGTTSFDLSTGCNNYDNQGHSQTDFP | SGRP (Liti et al., 2009),  Sanger Institute |
| YPS128 |  | GCPNLDFNWHMDQQNIMQYTLDVTSVSWVQDNTYQITIHVKGKENIDLKYLWSLKIIGVTGPKGTVQLYGYNENTYLIDNPTDFTATFEVYATQDVNSCQVWMPNFQIQFEYLQGSAAQYASSWKWGTTSFDLSTGCNNYDNQGHSQTDFP | SGRP (Liti et al., 2009),  Sanger Institute |
| NCYC110 |  | GCPNLDFNWHMDQQNIMEYTLDVTSVSWVQDNTYQITVHVKGKENIDLKYLWSLKIIGVTGPKGTVQLYGYNENTYLIDNPTDFTATFEVYATQDVNSCQVWMPNFQIQFEYLQGSAAQYASSWKWGTTSFDLSTGCNNYDNQGHSQTDFP | SGRP (Liti et al., 2009),  Sanger Institute |
| Y10 | PRJNA60201^3^ | GCPNLDFNWHMNQQNIMQYTLDVTSVSWVQDNTYQITIHVKGEENIDLKYLWSLKIIGVTGPKDTVQLYGYDEDTDWIDNPLVSRCDENTHLIDNPTDFTATFEVYATQDVNSCQVWMPNFQIQFEYLQGSAAQYASSWKWGTTSFDLSTGCNNYDNQGHSQTDFP | NCBI |
| YJSH1 | PRJNA72403^3^ | GCPNLDFNWHMNQQNIMQYTLDVTSVSWVQDNTYQITIHVKGEENIDLKYLSSLKIIGVTGPKDTVQLYGYHENTYLIDNPIDFVRCSENTHLIDNPTDFTATFEVYATQDVNSCQVWMPNFQIQFEYLQGSAAQYACSWEWGTTSFYLSTGCNNYDHQGHSQTDFP | NCBI |
| UC5 | PRJNA60197^3^ | GCPNLDFNWHMNQQNIMQYTLDVTSVSWVQDNTYQITIHVKGEENIDLKYLWSLKIIGVTGPKDTVQLYGYDEDTDWIDNPLVSRCDENTHLIDNPTDFTATFEVYATQDVNSCQVWMPNFQIQFEYLQGSAAEHACSWEWGTTSFYLSTGCDNYDNQGYSQTDFP | NCBI |
| SIHA_7^4^ | KX189102^3^ | GCPNLDFNWHMNQQTIMQYTLDVTSVSWVQDNTYQITIHVKGKENIDLNYLSSLKIIGVTGPKDTVQLYGCDENTYLIDNPTDFTATFEVYATQDVNSCQVWMPNFQIQFEYLQGSAAQYASSWKWGTTSFNLSTGCNNYDNQGHSQTDFP | This study |
| SIHA_Whitearome^4^ | KX189103^3^ | GCPNLDFNWHMDQQNIMQYTLDVTSVSWVQDNTYQITIHVKGKENIDLKYLWSLKIIGVTGPKGTVQLYGYNENTYLIDNPTDFTATFEVYATQDVNSCQVWMPNFQIQFEYLQGSAAQYASSWQWGTTSFDLSTGCNNYDNQGHSQTDFP | This study |
| Lalvin_R-HST^4^ | KX189104^3^ | GCPNLDFNWHMNQQTIMQYTLDVTSVSWVQDNTYQITIHVKGKENIDLKYLWSLKIIGVTGPKGTVQLYGYNENTYLIDNPTDFTATFEVYATQDVNSCQVWMPNFQIQFEYLQGSAAQYASSWKWGTTSFDLSTGCNNYDNQGHSQTDFP | This study |
| Uvaferm_SVG^4^ | KX189105^3^ | GCPNLDFNWHMNQQTIMQYTLDVTSVSWVQDNTYQITIHVKGKENIDLKYLWSLKIIGVTGPKGTVQLYGYNENTYLIDNPTDFTATFEVYATQDVNSCQVWMPNFQIQFEYLQGSAAQYASSWKWGTTSFDLSTGCNNYDNQGHSQTDFP | This study |
| Uvaferm_CEG^4^ | KX189106^3^ | GCPNLDFNWHMNQQTIMQYTLDVTSVSWVQDNTYQITIHVKGKENIDLNYLSSLKIIGVTGPKDTVQLYGCNENTYLIDNPTDFTATFEVYATQDVNSCQVWMPNFQIQFEYLQGSAAQYASSWKWGTTSFNLSTGCNNYDNQGHSQTDFP | This study |
| SSI2^4^ | KX189107^3^ | GCPNLDFNWHMNQQNIMQYTLDVTSVSWVQDNTYQITIHVKGEENIDLKYLSSLKIIGVTGPKDTVQLYGYDENTDWIDNPLVSRCDENTYLIDNPTDFTATFEVYATQDVNSCQVWMPNFQIQFEYLQGSAAEYACSWEWGTTSFYLSTGCNNYDNQGHSQTDFP | This study |
| SSI6^4^ | KX189108^3^ | GCPNLDFNWHMNQQNIMQYTLDVTSVSWVQDNTYQITIHVKGEENIDLKYLWSLKIIGVTGPKDTVQLYGYDEDTDWIDNPLVSRCDENTYLIDNPTDFTATFEVYATQDVNSCQVWMPNFQIQFEYLQGSAAEYACSWEWGTTSFYLSTGCDNYDNQGYSQTDFP | This study |
| YJM128^4^ | KX189109^3^ | GCPNLDFNWHMNQQNIMQYTLDVTSVSWVQDNTYQITIHVKGEENIDLEYLSSLKIIGVTGPKDTVQLYGYNEDTYLIDNPLVSRCDEYTYLIDNPTDFTATFEVYATQDVNSCQVWMPNFQIQFEYLQDIAPEDECSWEWGTTSFTLSTGCDNYDNQGYSQTDFP | This study |
| YJM222^4^ | KX189110^3^ | GCPNLDFNWHMNQQNIMQYTLDVTSVSWVQDNTYQITIHVKGKENIDLKYLWSLKIIGVTGPKGTVQLYGYNENTYLIDNPTDFTATFEVYATQDANSCQVWMPNFQIQFEYLQGSAAQYASTWTWGTTSFDLSTGCNNYDNQGHSQTDFP | This study |
| YJM308^4^ | KX189111^3^ | GCPNLDFNWHMNQQNIMQYTLDVTSVSWVQDNTYQITIHVKGKENIDLKYLWSLKIIGVTGPKGTVQLYGYNENTYLIDNPTDFTATFEVYATQDVNSCQVWMPNFQIQFEYLQGSAAQYACSWEWGTTSFDLSTGCNNYDNQGHSQTDFP | This study |
| YJM309^4^ | KX189112^3^ | GCPNLDFNWHMNQQNIMQYTLDVTSVSWVQDNTYQITIHVKGEENIDLEYLSSLKIIGVTGPKDTVQLYGYNEDTYLIDNPLVSRCDEYTYLIDNPTDFTATFEVYATQDVNSCQVWMPNFQIQFEYLQDIAPEDECSWEWGTTSFTLSTGCDNYDNQGYSQTDFP | This study |
| YJM311^4^ | KX189113^3^ | GCPNLDFNWHMNQQNIMQYTLDVTSVSWVQDNTYQITIHVKGKENIDLKYLWSLKIIGVTGPKGTVQLYGYNENTYLIDNPTDFTATFEVYATQDVNSCQVWMPNFQIQFEYLQGSAAQYASSWKWGTTSFDLSTGCNNYDNQGHSQTDFP | This study |
| YJM312^4^ | KX189114^3^ | GCPNLDFNWHMNQQNIMQYTLDVTSVSWVQDNTYQITIHVKGEENIDLKYLWSLKIIGVTGPKDTVQLYGYDEDTDWIDNPLVSRCDENTHLIDNPTDFTATFEVYATQDVNSCQVWMPNFQIQFEYLQGSAAQYASSWQWGTTSFDLSTGCNNYDNQGHSQTDFP | This study |
| SSI3^4^ | KX189115^3^ | GCPNLDFNWHMNQQNIMQYTLDVTSVSWVQDNTYQITIHVKGKENIDLKYLWSLKIIGVTGPKGTVQLYGYNENTYLIDNPTDFTATFEVYATQDVNSCQVWMPNFQIQFEYLQGSAAQYASSWKWGTTSFDLSTGCNNYDNQGHSQTDFP | This study |
| SSI4^4^ | KX189116^3^ | GCPNLDFNWHMNQQSIMQYTLDVISVSWVQDDTYQITIHVEGKENIDLNYLSSLKIIDVTGPEDTVQLYGCNEEDTYLIDNPTDFTATFEVYATQDVNSGQVWMSDFQIQFEYLQDSAAQYASSWEWGATSFGLSTGCNNYDDQGHSQTDFP | This study |
| SSI9^4^ | KX189117^3^ | GCSNLDFNWHMNQQNIMQYTLDVTSVSWVQDNTYQITIHVKGEENIDLNYLSSLKIIGVTGPKDTVQLYGYDENTYLIDNPLVSRYDENTYLIDNPTDFTATFEVYATQDVNSCQVWMPDFQIQFEYLQDSAAQYASSWEWGTTSFTLSTGCDNYDDQGYSQTDFP | This study |
| A6^4^ | KX189118^3^ | GCPNLDFNWHMNQQNIMQYTLDVTSVSWVQDNTYLITIHVKGKENIDLNYLSSLKIIGLTGPEDTVQLYGCNEYTYLIDNPTDFTATFEVYATPDVNSCQVWMPDFQIQFEYLQDSAAQYASSWKWGTTSFTLSTGCNNYDSQGHSQTDFP | This study |
| A18^4^ | KX189119^3^ | GCPNLDFNWHMNQQNIMQYTLDVTSVSWVQYNTYQITIHVKGKENIDLKYLSSLKIIGLTGPKDTVQLYGCNENTYLIDNPTDFTATFEVYATQDVNSCQVWMPNFQIQFKYLQGRAAQYASSWKWGTTSFDLSTGCNNYDNQGHSLTDFP | This study |
| KVL012^4^ | KX189120^3^ | GCPNLDFNWHMNQQTIMQYTLDVTSVSWVQDNTYQITIHVKGKENIDLKYLWSLKIIGVTGPKGTVQLYGYNENTYLIDNPTDFTATFEVYATQDVNSCQVWMPDFQIQFEYLQDSAAQYASSWKWGTTSFALSTGCNNYDNQGHSQTDFP | This study |
| C1^4^ | KX189121^3^ | GCPNLDFNWHMNQQNIMQYTLDVTSVSWVQYNTYQITIHVKGKENIDLKYLSSLKIIGLTGPKDTVQLYGCNENTYLIDNPTDFTATFEVYATQDVNSCQVWMPNFQIQFKYLQGRAAQYASSWKWGTTSFDLSTGCNNYDSQGHSQTDFP | This study |
| *S. paradoxus*  CBS432 |  | GCPNLDFNWHMDQQNIMEYTLDVTSVSWVQDNTYQITIHVKGKENIDLKYLWSLKVIGVTGPKGTVQLYGTNENTYLIDNPTDFTATFEVYATQDVNSCQVWMPNFQIQFEYLQGTAAQYASTWKWGTTSFDLSTGCNNYDNQGHSQTDFP | SGRP (Liti et al., 2009),  Sanger Institute |
| *S. kudriavzevii*  IFO1802 | PRJNA1442^3^ | GCPSLDFNWHMNQQNIMQYTLDVTSVSWVQDNTYQIQIHVKGKENIDLKYLWSLKVIGVNGPKGTVQLYGFNENTFLIDNPTDFTATFEVYATQDVNSCQVWMPNFQIQFEYLQGSAAQYSSTWEWGTTSFDLSTGCNNYDNQGHSQTDFP | NCBI |
| *S. bayanus*  MCYC623 | PRJNA1443^3^ | GCPSLNFNWHMDQQNIMEYTLDVTSVSWVQDNTYQIQIHVKGKENIDLKYLWSLKVIGVTGPQGTVQLYGTNENTYLIDNPTDFTATFEVYATQDVNSCQVWMPNFQIQFEYLQGTAAQYASTWEWGTTSFDLSTGCNNYDNQGHSQTDFP | NCBI |
| *S. mikatae*  IFO1815T | PRJNA374^3^ | GCPSLDFNWHMNQQNIMQYTLDVTSVSWVRDNTYQIQIHVKGKENIDLKYLWSLKVIGVNGPKGTVQLYGHNENTYLIDNPTDFTATFEVYATQDVDSCQVWMPNFQIQFEYLQGSAAQYASSGKWGTTSFDLSTGSNNYDNQGHSQTDFP | NCBI |
| *S. arboricolus* | PRJNA88533^3^ | GCPSLNFDWHMDQQNIMEYTLDVTSVSWVQDNTYQIQIHVKGKETIDIKYLWSLKVIGVSGPQGTVQLYGHNENVYLIDNPTDFTATFEVYATQDVNSCQVWMPNFQIQFEYLEGSTAQYSSTWKWGKSAFDLSTGCNNYDNQGHSQTDFP | NCBI |
| *K. pastoris* | C4R2D7_A^2^ | CYANQWETTFPPSDIKITGATWVQDNIYDVTLSYEAESLELENLTELKIIGLNSPTGGTKLVWSLNSKVYDIDNPAKWTTTLRVYTKSSADDCYVEMYPFQIQVDWCEAGASTDGCSAWKWPKSYDYDIGCDNMQDGVSRKHHP | UniProtKB |
| *K. pastoris* | C4R2D7_B^2^ | CYADQWETTFPPSDIKITGATWVEDNIYDVTLSYEAESLELENLTELKIIGLNSPTGGTKVVWSLNSGIYDIDNPAKWTTTLRVYTKSSADDCYVEMYPFQIQVDWCEAGASTDGCSAWKWPKSYDYDIGCDNMQDGVSRKHHP | UniProtKB |
| *C. lusitaniae* | C4XZ24_A^2^ | ACPNQKFTFHAQVVNFPQATITVTDPSDNGDGTWDVTINFNADATMSLKSLSELKILSLSKTYFLYSYNLKVDNINNPGSWSQRVTVTPRSVGDYKTCMPQFTIQYDWCSAGVTDWSECQNWKYQGSYDYITGCDNFDQSTGFSQKDAP | UniProtKB |
| *C. lusitaniae* | C4XZ24_B^2^ | ACPNQKFTFHAQVVNFPQATITVTDPSDNGDGTWDVTINFNAVATMSLKSLSELKILSLSKTYFLYSYNLKVDNINNPGSWSQRVTVTPRSVGNYKTCMPQFTIQFDWCSAGVTDQSECQNWKYQGSYDYITGCDNFDQSTGFSQKDAP | UniProtKB |
| *C. lusitaniae* | C4XZ24_C^2^ | ACPNQKFTFHAQVVNFPQATITVTDPSDNGDGTWDVTINFNAVATMSLKSLSELKILSLSKTYFLYSYNLKVDNINNPGSWSQRVTVTPRSVGNYKTCMPQFTIQYDWCSAGVTDWSECQNWKYQGSYDYITGCDNFDQSTGFSQKDAP | UniProtKB |
| *K. lactis* | Q6CPZ4_A^2^ | GCPCLDFSFRSQNTRTMPYNIELENVKWVESNIYTVTLHVTGQKQIPLKSLWSLKIIGVNSPDGSTFQLFGYNEKTYLIDNPTDWTATFRVYGQADSNDPSIVWMPTFQIQYEYCQGSADCSDWSYGTTTFDLITGCNNYDNYKRSQTDAG | UniProtKB |
| *K. lactis* | Q6CPZ4_B^2^ | GCPCLDFSFHSQNTGTMQYSIVPEEVNWVQDNIYTVTLHVTGQKQIPLKSLWSLKIIGVNSPDGSTFQLFGYNEKTYLIDNPTDWTATFRVYGQADSNDPSIVWMPTFQIQYEYCQGSADCSDWSYGTTTFDLITGCNNYDNYKRSQTDAG | UniProtKB |
| *S. passalidarum* | G3AVC2_A^2^ | ICDSPFGALSKANNQPELKIFEFKSIKWIEDNFYEVMIEFEIDGNSYTESELRAIYIFSLQTPDYYLGSVELFVESWEQNLLGDSPFHFYFTWVMEAEDIDQLTCTTPFQVYYDWDTYFATYQHGCFSDELTDLPAQCWDKPNFAETSSQQNLETSSQQNLE | UniProtKB |
| *S. passalidarum* | G3AVC2_B^2^ | IPIGAHALSESTYPKVKTFNFESIKFIQDDLYEVTLEFEIEDTIPKSELHAVFAYYLQTPDDYLSSIQLDAYVGNSPYHFYLVWIMKTQNIDQYICTTPFTIEYNWNGIPFSYTHGCL | UniProtKB |
| *T. delbrueckii* | G8ZQZ3^2^ | ACPGLDFKWRSTYYGIMNYDMNVIGVNYLGKDSYEVTIHVVGDKQIPLKFLYSLSISNIGGPDRIVPLHHCDKGINKIDNPTDFTATFVVNSRPDLFGRVWMPDFKLDFEYVLGPARQYAKEWKWGKTSFSLASGCDLADFWGRSYADFP | UniProtKB |
| *M. guilliermondii* | A5DGW9^2^ | AGLLQQRATQQPPKAVFQVSAVAHVEANLFKVTLDFETAASAQLFQSFANSAKSAKVTGLGSDLQSDAVVVGADASTGIDNLFAFSVSVLIEASIHNGLFCLPDGLAIELDLDLDVNTDVGKLWQEYFPQNIKYALDQNFQDAKQFTPSKRQD | UniProtKB |

^1^For *S. cerevisiae* strains, only names are shown.

^2^UniProt KnowledgeBase (UniProtKB) identification number followed by relative position of Flo11-type domain (A, B or C) for proteins with multiple Flo11A domains.

^3^GenBank database Gene, Genome or BioProject identification number (www.ncbi.nlm.nih.gov).

^4^Strains were described (Liti et al., 2009, Klingberg et al., 2008) and used in the current study for isolation and sequencing of *FLO11A* encoding DNA fragments.

**References**

KLINGBERG, T. D., LESNIK, U., ARNEBORG, N., RASPOR, P. & JESPERSEN, L. 2008. Comparison of *Saccharomyces cerevisiae* strains of clinical and nonclinical origin by molecular typing and determination of putative virulence traits. *FEMS Yeast Res,* 8**,** 631-40.

LITI, G., CARTER, D. M., MOSES, A. M., WARRINGER, J., PARTS, L., JAMES, S. A., DAVEY, R. P., ROBERTS, I. N., BURT, A., KOUFOPANOU, V., TSAI, I. J., BERGMAN, C. M., BENSASSON, D., O'KELLY, M. J., VAN OUDENAARDEN, A., BARTON, D. B., BAILES, E., NGUYEN, A. N., JONES, M., QUAIL, M. A., GOODHEAD, I., SIMS, S., SMITH, F., BLOMBERG, A., DURBIN, R. & LOUIS, E. J. 2009. Population genomics of domestic and wild yeasts. *Nature,* 458**,** 337-41.
