# Supplementary material for: Kin discrimination in social yeast is mediated by cell surface receptors of the Flo11 adhesin family
Source: eLife. 2020 Apr 14;9:e55587. doi: 10.7554/eLife.55587 (PMC7156268; doi:10.7554/eLife.55587)
Supplement: Supplementary file 2. [file elife-55587-supp2.docx]

**Supplementary File 2**

**Yeast strains used in this study.**

| **Strain** | **Name in text and figures** | **Relevant genotype** | **Reference or source** |
| --- | --- | --- | --- |
| RH2662 | - | Σ1278b *MAT****a*** *flo11∆::kanR ura3-52* | (Braus et al., 2003) |
| RH2681 | - | Σ1278b *MAT****a*** *flo11∆::kanR ura3-52 trp1::hisG* | (Herzog et al., 2013) |
| YHUM3007 | - | *P_TDH3_-RFP-T_CYC1_ ura3-52* in RH2681 | This study |
| YHUM3008 | - | *P_TDH3_-GFP-T_CYC1_  ura3-52* in RH2681 | This study |
| YHUM3014 | ScFlo11A | *P_FLO11_Σ1278b_- FLO11_S288c(P08640)_- T_FLO11_S288c(P08640)_* in YHUM3007 | This study |
| YHUM3015 | ΔA | *P_FLO11_Σ1278b_- FLO11_S288c(P08640)_^M1-G30^-FLO11_S288c(P08640)_^T208-L1360^- T_FLO11_S288c(P08640)_* in YHUM3007 | This study |
| YHUM3016 | Sc^Σ^Flo11A | *P_FLO11_Σ1278b_- FLO11_S288c(P08640)_^M1-G30^- FLO11A_Σ1278b(E9P9G2)_^S31-C220^-FLO11_S288c(P08640)_^T208-L1360^- T_FLO11_S288c(P08640)_* in YHUM3007 | This study |
| YHUM3017 | KpFlo11A | *P_FLO11_Σ1278b_- FLO11_S288c(P08640)_^M1-G30^-FLO11A _K. pastoris(C4R2D7_A)_^P29-W184^-FLO11_S288c(P08640)_^T208-L1360^-T_FLO11_S288c(P08640)_* in YHUM3007 | This study |
| YHUM3018 | ClFlo11A | *P_FLO11_Σ1278b_- FLO11_S288c(P08640)_^M1-G30^-FLO11A_C. lusitaniae(C4XZ24_A)_^V20-W175^-FLO11_S288c(P08640)_^T208-L1360^-T_FLO11_S288c(P08640)_* in YHUM3007 | This study |
| YHUM3019 | ScFlo11A | *P_FLO11_Σ1278b_- FLO11_S288c(P08640)_- T_FLO11_S288c(P08640_* in YHUM3008 | This study |
| YHUM3020 | ΔA | *P_FLO11_Σ1278b_- FLO11_S288c(P08640)_^M1-G30^-FLO11_S288c(P08640)_^T208-L1360^- T_FLO11_S288c(P08640)_* in YHUM3008 | This study |
| YHUM3021 | Sc^Σ^Flo11A | *P_FLO11_Σ1278b_- FLO11_S288c(P08640)_^M1-G30^- FLO11A_Σ1278b(E9P9G2)_^S31-C220^-FLO11_S288c(P08640)_^T208-L1360^- T_FLO11_S288c(P08640)_* in YHUM3008 | This study |
| YHUM3022 | KpFlo11A | *P_FLO11_Σ1278b_- FLO11_S288c(P08640)_^M1-G30^-FLO11A _K. pastoris(C4R2D7_A)_^P29-W184^-FLO11_S288c(P08640)_^T208-L1360^-T_FLO11_S288c(P08640)_* in YHUM3008 | This study |
| YHUM3023 | ClFlo11A | *P_FLO11_Σ1278b_- FLO11_S288c(P08640)_^M1-G30^-FLO11A_C. lusitaniae(C4XZ24_A)_^V20-W175^-FLO11_S288c(P08640)_^T208-L1360^-T_FLO11_S288c(P08640)_* in YHUM3008 | This study |

**References**

BRAUS, G. H., GRUNDMANN, O., BRÜCKNER, S. & MÖSCH, H.-U. 2003. Amino acid starvation and Gcn4p regulate adhesive growth and *FLO11* gene expression in *Saccharomyces cerevisiae*. *Mol Biol Cell,* 14**,** 4272-84.

HERZOG, B., POPOVA, B., JAKOBSHAGEN, A., SHAHPASANDZADEH, H. & BRAUS, G. H. 2013. Mutual cross talk between the regulators Hac1 of the unfolded protein response and Gcn4 of the general amino acid control of Saccharomyces cerevisiae. *Eukaryot Cell,* 12**,** 1142-54.
