# Supplementary material for: Kin discrimination in social yeast is mediated by cell surface receptors of the Flo11 adhesin family
Source: eLife. 2020 Apr 14;9:e55587. doi: 10.7554/eLife.55587 (PMC7156268; doi:10.7554/eLife.55587)
Supplement: Supplementary file 3. [file elife-55587-supp3.docx]

**Supplementary File 3**

**Plasmids used in this study.**

| **Plasmid** | **Name in text and figures** | **Relevant genotype^1,2^** | **Reference or source** |
| --- | --- | --- | --- |
| BHUM2200 | ΔA | *P_PGK1_-FLO11_S288c(P08640)_^M1-G30^-BamHI-PauI-FLO11_S288c(P08640)_^T208-L1360^-T_FLO11_S288c(P08640)_, CEN, URA3* | (Kraushaar et al., 2015) |
| BHUM2422 | ScFlo11A | *FLO11A_S288c(P08640)_^S31-G207^* in BHUM2200 | (Kraushaar et al., 2015) |
| BHUM2612 | Y111D Y113D Y118D | BHUM2422 with *Y111D Y113D Y118D* | (Kraushaar et al., 2015) |
| BHUM2117 | Y111A Y113A Y118A | BHUM2422 with *Y111A Y113A Y118A* | (Kraushaar et al., 2015) |
| BHUM2413 | W94D Y162D | BHUM2422 with *W94D Y162D* | This study |
| BHUM2987 | Y92D Y154D | BHUM2422 with *Y92D Y154D* | This study |
| BHUM2415 | W166D W168D | BHUM2422 with *W166D W168D* | (Kraushaar et al., 2015) |
| BHUM2754 | W166A W168A | BHUM2422 with *W166A W168A* | This study |
| BHUM2419 | Y133D W144D Y196D | BHUM2422 with *Y133D W144D Y196D* | This study |
| BHUM2989 | W70D Y76D | BHUM2422 with *W70D Y76D* | This study |
| BHUM2753 | W51D Y182D | BHUM2422 with *W51D Y182D* | This study |
| BHUM2756 | Y133A W144A Y196A | BHUM2422 with Y133A W144A Y196A | This study |
| BHUM2758 | Y111A Y113A Y118A Y133A W144A Y196A | BHUM2422 with *Y111A Y113A Y118A Y133A W144A Y196A* | This study |
| BHUM3000 | W70A Y76A Y92A Y111A Y113A Y118A Y133A W144A Y154A W166A W168A Y196A | BHUM2422 with *W70A Y76A Y92A Y111A Y113A Y118A Y133A W144A Y154A W166A W168A Y196A* | This study |
| BHUM2884 | ∆3_10_-helix | BHUM2422 with *∆Q156-S165::GSG* | This study |
| BHUM2883 | ∆α-helix | BHUM2422 with *∆N50-57N::GSGS* | This study |
| BHUM2955 | ScFlo11A^Σins^ | BHUM2422 with *∆N116::NTDWIDNPLVSRCDEN S164C* | This study |
| BHUM2223 | Sc^Σ^Flo11A | *FLO11A_Σ1278b(E9P9G2)_^S31-C220^* in BHUM2200 | This study |
| BHUM2920 | STREPII-SpFlo11A | *STREP II-FLO11A_S. paradoxus_CBS432_^S31-G208^* in BHUM2200 | This study |
| BHUM2923 | STREPII-KlFlo11A | *STREP II-FLO11A_K. lactis(Q6CPZ4_A)_^I41-W197^* in BHUM2200 | This study |
| BHUM2906 | STREPII-TdFlo11A | *STREP II-FLO11A_T. delbrueckii(G8ZQZ3)_^N58-W212^* in BHUM2200 | This study |
| BHUM2889 | KpFlo11A | *FLO11A_K. pastoris(C4R2D7_A)_^P29-W184^* in BHUM2200 | This study |
| BHUM2921 | STREPII-KpFlo11A | *STREP II-FLO11A_K. pastoris(C4R2D7_A)_^P29-W184^* in BHUM2200 | This study |
| BHUM2890 | ClFlo11A | *FLO11A_C. lusitaniae(C4XZ24_A)_^V20-W175^* in BHUM2200 | This study |
| BHUM2922 | STREPII-ClFlo11A | *STREP II-FLO11A_C. lusitaniae(C4XZ24_A)_^V20-W175^* in BHUM2200 | This study |
| BHUM2907 | STREPII-MgFlo11A | *STREP II-FLO11A_M. guilliermondii(A5DGW9)_^I65-L228^* in BHUM2200 | This study |
| BHUM1578 | ScFlo11A | *ScFlo11A^F22-S211^* in pET28a(+) | (Kraushaar et al., 2015) |
| BHUM3113 | KpFlo11A | *KpFlo11A^S23-189^* in pET28a(+) | This study |
| YIplac211 | - | *URA3 AmpR* | (Gietz and Sugino, 1988) |
| BHUM0778 | - | *P_FLO11_Σ1278b_- FLO11_Σ1278b(E9P9G2)_-T_FLO11_Σ1278b_ CEN URA3* | (Braus et al., 2003) |
| BHUM3353 | - | *P_FLO11_Σ1278b_- FLO11_S288c(P08640)_- T_FLO11_S288c(P08640)_* in YIplac211 | This study |
| BHUM3354 | - | *P_FLO11_Σ1278b_- FLO11_S288c(P08640)_^M1-G30^-FLO11_S288c(P08640)_^T208-L1360^- T_FLO11_S288c(P08640)_* in YIplac211 | This study |
| BHUM3356 | - | *P_FLO11_Σ1278b_- FLO11_S288c(P08640)_^M1-G30^- FLO11A_Σ1278b(E9P9G2)_^S31-C220^-FLO11_S288c(P08640)_^T208-L1360^- T_FLO11_S288c(P08640)_* in YIplac211 | This study |
| BHUM3359 | - | *P_FLO11_Σ1278b_- FLO11_S288c(P08640)_^M1-G30^-FLO11A _K. pastoris(C4R2D7_A)_^P29-W184^-FLO11_S288c(P08640)_^T208-L1360^-T_FLO11_S288c(P08640)_* in YIplac211 | This study |
| BHUM3360 | - | *P_FLO11_Σ1278b_- FLO11_S288c(P08640)_^M1-G30^-FLO11A_C. lusitaniae(C4XZ24_A)_^V20-W175^-FLO11_S288c(P08640)_^T208-L1360^-T_FLO11_S288c(P08640)_* in YIplac211 | This study |
| pRS304 | - | *TRP1 AmpR* | (Sikorski and Hieter, 1989) |
| pDS90 | - | *P_TDH3_-* *tagRFP-AtLOV2-cODC1* | (Usherenko et al., 2014) |
| pYM12 |  | *yEGFP-kanMX4* | (Knop et al., 1999) |
| BHUM3349 | - | *P_TDH3_-tagRFP-T_CYC1_* in pRS304 | This study |
| BHUM3350 | - | *P_TDH3_-yEGFP-T_CYC1_* in pRS304 | This study |

^1^UniProt KnowledgeBase identification numbers of *FLO11A* domain bearing proteins are shown in parentheses, when available.

^2^For proteins carrying multiple Flo11A domains, the relative position (A, B or C) of the domain used is indicated after the UniProt number.

^3^For *S. paradoxus*, *FLO11A* from strain CBS432 was used.

**References**

BRAUS, G. H., GRUNDMANN, O., BRÜCKNER, S. & MÖSCH, H.-U. 2003. Amino acid starvation and Gcn4p regulate adhesive growth and *FLO11* gene expression in *Saccharomyces cerevisiae*. *Mol Biol Cell,* 14**,** 4272-84.

GIETZ, R. D. & SUGINO, A. 1988. New yeast-*Escherichia coli* shuttle vectors constructed with in vitro mutagenized yeast genes lacking six-base pair restriction sites. *Gene,* 74**,** 527-534.

KNOP, M., SIEGERS, K., PEREIRA, G., ZACHARIAE, W., WINSOR, B., NASMYTH, K. & SCHIEBEL, E. 1999. Epitope tagging of yeast genes using a PCR-based strategy: more tags and improved practical routines. *Yeast,* 15**,** 963-72.

KRAUSHAAR, T., BRÜCKNER, S., VEELDERS, M., RHINOW, D., SCHREINER, F., BIRKE, R., PAGENSTECHER, A., MÖSCH, H.-U. & ESSEN, L.-O. 2015. Interactions by the Fungal Flo11 Adhesin Depend on a Fibronectin Type III-like Adhesin Domain Girdled by Aromatic Bands. *Structure,* 23**,** 1005-17.

SIKORSKI, R. S. & HIETER, P. 1989. A system of shuttle vectors and yeast host strains designed for efficient manipulation of DNA in *Saccharomyces cerevisiae*. *Genetics,* 122**,** 19-27.

USHERENKO, S., STIBBE, H., MUSCO, M., ESSEN, L. O., KOSTINA, E. A. & TAXIS, C. 2014. Photo-sensitive degron variants for tuning protein stability by light. *BMC Syst Biol,* 8**,** 128.
