# Supplementary material for: Kin discrimination in social yeast is mediated by cell surface receptors of the Flo11 adhesin family
Source: eLife. 2020 Apr 14;9:e55587. doi: 10.7554/eLife.55587 (PMC7156268; doi:10.7554/eLife.55587)
Supplement: Supplementary file 4. [file elife-55587-supp4.docx]

**Supplementary File 4**

**Statistics for crystal structure data collection, processing, and refinement.**

| **Data collection & processing** | ***Kp*Flo11A**  **Crystal form I**  **(5FV5)** | ***Kp*Flo11A**  **Crystal form II**  **(5FV6)** |
| --- | --- | --- |
| X-ray source | MX-14-1, BESSY II,  Berlin, Germany | MX-14-1, BESSY II,  Berlin, Germany |
| detector | Pilatus 6M | Pilatus 6M |
| wavelength (Å) | 0.977790 | 0.977790 |
| space group | *P*2_1_2_1_2_1_ | *P*2_1_ |
| cell dimensions (*a,b,c* Å)  Angles (°) | 35.54 58.52 76.55  90 90 90 | 37.44 58.68 85.27  90 96.56 90 |
| resolution (Å) | 46.49 - 1.40 (1.48 - 1.40) | 42.36 - 2.00 (2.07 - 2.00) |
| total reflections | 284209 | 76825 |
| multiplicity | 6.7 | 3.2 |
| unique reflections | 42777 | 24319 |
| R_merge_ (%) | 5.6 (45.6) | 9.1 (51.9) |
| Completeness overall (%) | 96.4 | 97.5 |
| Completeness inner shell (%) | 98.0 | 98.8 |
| Completeness outer shell (%) | 94.7 | 98.3 |
| *I/σ(I)* | 20.95 (4.33) | 9.96 (2.72) |
| mosaicity (°) | 0.11 | 0.34 |
| Wilson B-factor (Å^2^) | 12.0 | 23.8 |
| CC (1/2) (%) | 89.6 | 80.2 |
|  |  |  |
| **Refinement statistics** |  |  |
| resolution (Å) | 46.49-1.40 | 42.36-2.00 |
| R_factor_, R_free_ (%) | 13.4, 17.9 | 22.4, 26.6 |
| reflections (working, test set) | 29332, 1581 | 24300, 1239 |
| r.m.s.d. from ideal: |  |  |
| bond lengths (Å) | 0.0121 | 0.0115 |
| bond angles (°) | 1.659 | 1.485 |
| total number of atoms | 1658 | 3114 |
| mean B value (Å^2^) | 16.9 | 27.6 |
