# Supplementary material for: Kin discrimination in social yeast is mediated by cell surface receptors of the Flo11 adhesin family
Source: eLife. 2020 Apr 14;9:e55587. doi: 10.7554/eLife.55587 (PMC7156268; doi:10.7554/eLife.55587)
Supplement: Supplementary file 5. [file elife-55587-supp5.docx]

**Supplementary File 5**

**Structural analysis of KpFlo11A.**

Crystallization of KpFlo11A was performed at a protein concentration of 70 mg/ml in HEPES buffer pH 7.5. Crystal growth was observed after two days in two conditions (see experimental procedure) and could be reproduced in hanging drops with 35 mg/ml. The structure was solved by molecular replacement using the ScFlo11A structure as template for model building with the KpFlo11A sequence (Supplementary File 4). The KpFlo11A domain was crystallized in an orthorhombic space group (*P*2_1_2_1_2_1_) with one molecule per asymmetric symmetry unit, and in a monoclinic space group (*P*2_1_) with two molecules per asymmetric symmetry unit. In space group *P*2_1_ the crystal contacts within the antiparallel orientated dimer are mainly caused by polar interactions between six residues and differs thereby from the ScFlo11A-ScFlo11A contacts, which are mainly found to depend on aromatic residues. To further investigate the physiological relevance of the dimer an interface analysis using the PDBePISA (Krissinel and Henrick, 2007) server was conducted. The surface area of this interface is 437 Å^2^ with a Complex Formation Significance Score (CSS) of 0.00. This lack of significance implies that the interface is a result of crystal packing, but not of physiologically relevant dimer formation. Likewise, the orthorhombic crystal form lacks any KpFlo11A-KpFlo11A interaction that has a chance to exist outside the packing context. For comparison, the interface between the modules Ig1 and Ig2 of the classical neural cell adhesion molecule (NCAM) has an extent of 1594 Å^2,^(Soroka et al., 2003). NCAM mediates cell-cell recognition and adhesion via a well-defined homophilic interaction (Kasper et al., 2000) and thus this dimer was found to be of high physiological relevance.

The topology of KpFlo11A resembles the structure of ScFlo11A, but has several distinct features. KpFlo11A harbors three β-sheets but only minor α-helical elements. β-sheet I is formed by β-strands 2, 3 and 6, β-sheet II by β strands 4, 5, 8 and 9, and β-sheet III by β-strands 1, 7 and 10. β-sheet one and two form a beta-sandwich, which constitutes the fibronectin type III domain, while β-sheet III forms the neck-like subdomain that presents a junction to the B1 and B2 regions. As the neck-like subdomain represents a hinge to the downstream B regions, it probably provides a rigid exposition of the Flo11A domains to the cellular environment. The region that is apical to the FNIII domain consists mainly of longer loop regions. Despite a sequence identity between KpFlo11A and ScFlo11A of only 32%, their overall structures show a high degree of similarity with an r.m.s.d. of 1.01 Å for 102 Cα atoms after superposition (Figure 1–figure supplement 1 and Figure 4–figure supplement 2). As expected, the FNIII domain shows the highest degree of similarity (0.70 Å for 61 Cα atoms derived from the β-sheet), whereas the rest of KpFlo11A, the neck-like subdomain and the apical regions, significantly deviate from ScFlo11A.

Apart from the N- and C-terminal ends, the neck region shows only local structural differences given that its three disulfide bridges for linkage to the FNIII core are well conserved among most Flo11 orthologs including ScFlo11 (C37/C167, C129/C192 and C28/C188). For example, β6 from sheet I continues partly into the neck region so that the latter consists of only three β strands instead of four as in ScFlo11A. Interestingly, the linker between the neck subdomain and the FNIII core lacks the α-helical segment of the ScFlo11A domain and adopts a distorted conformation. Likewise, the apical region shows several structural deviations, particularly the region between β8 and β9. Here, an additional disulfide bridge between C143 and C152 that is missing in ScFlo11A causes the replacement of the 3_10_-helix in ScFlo11A by an irregular, disulfide-bridge stabilized loop region.

A hallmark of the structure of ScFlo11A is its high degree of solvent-exposed aromatic residues, notably 6 of its 7 tryptophans and 8 of its 11 tyrosines. These residues were found to be responsible for homophilic binding (Kraushaar et al., 2015). All of these residues were found to be parts of two surface-exposed bands and further surrounded by acidic residues. The latter ensures that homophilic adhesion only occurs at lower pH as given by the natural environmental conditions of *S. cerevisiae*. Our analysis of the KpFlo11A structure shows an analogous surface patterning of aromatic residues. Here, 6 of 8 tryptophans and 9 of 10 tyrosines are surface-exposed and cluster in two bands (Figure 4 and Figure 4–figure supplement 2). These bands, I and II, are found at the corresponding surface regions of ScFlo11A, although their constituting structural elements, especially the apical regions and the linker between neck-like subdomain and FNIII domain, differ. Accordingly, half of the surface-exposed aromatic residues were found to be either conserved (W58, Y64, Y106, Y121, W155, W157, W184) or altered from tyrosines to tryptophans (W99, W142). The other half has mostly hydrophobic counterparts in the ScFlo11A structure (V, L, F, P). Given a low isoelectric point (pI) of 5.5 that resembles the ScFlo11A domain we examined the distribution of acidic residues on the surface of KpFlo11A. As expected this domain has a high prevalence of surface-exposed acidic residues, namely nine glutamates and 13 aspartates. The surface distribution shows that these acidic residues co-cluster similarly with the two aromatic bands (data not shown) as in the ScFlo11A domain. Here, only one third is conserved between these two Flo11 orthologs (D61, E73, E76, D109, D141, D162, D172), the other are derived from different types of surface-exposed residues. Interestingly, besides the high occurrence of solvent exposed aromatic and acidic residues we also found an increased prevalence of adjacent proline residues. There are 5 residues in ScFlo11A, but 10 in KpFlo11A. Generally, this residue type has often a structural role, e.g. in stabilizing bends and turns. However, when analyzing ScFlo11-mediated adhesion to polystyrene surfaces, Mortensen and coworkers showed by phage display techniques that not only tryptophans, but also co-occurring prolines are major factors for surface adhesion (Mortensen et al., 2007). Indeed, apart from P29 all prolines are adjacent to or pack with tryptophans and tyrosines. Seven of these prolines are (partly) surface-exposed so that they may contribute either to hydrophobic interaction or steric stabilization of the aromatic side chains.

**References**

KASPER, C., RASMUSSEN, H., KASTRUP, J. S., IKEMIZU, S., JONES, E. Y., BEREZIN, V., BOCK, E. & LARSEN, I. K. 2000. Structural basis of cell-cell adhesion by NCAM. *Nat Struct Biol,* 7**,** 389-93.

KRAUSHAAR, T., BRÜCKNER, S., VEELDERS, M., RHINOW, D., SCHREINER, F., BIRKE, R., PAGENSTECHER, A., MÖSCH, H.-U. & ESSEN, L.-O. 2015. Interactions by the Fungal Flo11 Adhesin Depend on a Fibronectin Type III-like Adhesin Domain Girdled by Aromatic Bands. *Structure,* 23**,** 1005-17.

KRISSINEL, E. & HENRICK, K. 2007. Inference of macromolecular assemblies from crystalline state. *J Mol Biol,* 372**,** 774-97.

MORTENSEN, H. D., DUPONT, K., JESPERSEN, L., WILLATS, W. G. & ARNEBORG, N. 2007. Identification of amino acids involved in the Flo11p-mediated adhesion of *Saccharomyces cerevisiae* to a polystyrene surface using phage display with competitive elution. *J Appl Microbiol,* 103**,** 1041-7.

SOROKA, V., KOLKOVA, K., KASTRUP, J. S., DIEDERICHS, K., BREED, J., KISELYOV, V. V., POULSEN, F. M., LARSEN, I. K., WELTE, W., BEREZIN, V., BOCK, E. & KASPER, C. 2003. Structure and interactions of NCAM Ig1-2-3 suggest a novel zipper mechanism for homophilic adhesion. *Structure,* 11**,** 1291-301.
