# Supplementary material for: Kin discrimination in social yeast is mediated by cell surface receptors of the Flo11 adhesin family
Source: eLife. 2020 Apr 14;9:e55587. doi: 10.7554/eLife.55587 (PMC7156268; doi:10.7554/eLife.55587)
Supplement: Supplementary file 6. [file elife-55587-supp6.docx]

**Supplementary File 6**

**Quantification of Flo11A protein amounts at the cell surface by immunofluorescence microscopy.**

| **Flo11 variant as named in text and figures** | **Fluorescence relative to ScFlo11A** |
| --- | --- |
| ScFlo11 | 100%^1^ |
| Y111D Y113D Y118D | 92%^1^ |
| Y111A Y113A Y118A | 86%^1^ |
| W94D Y162D | 95%^1^ |
| Y92D Y154D | 87%^1^ |
| W166D W168D | 74%^1^ |
| W166A W168A | 98%^1^ |
| Y133D W144D Y196D | 93%^1^ |
| Y133A W144A Y196A | 73%^1^ |
| W70D Y76D | 72%^1^ |
| W51D Y182D | 84%^1^ |
| Y111A Y113A Y118A Y133A W144A Y196A | 60%^1^ |
| W70A Y76A Y92A Y111A Y113A Y118A Y133A W144A Y154A W166A W168A Y196A | 63%^1^ |
| ∆3_10_-helix | 99%^1^ |
| ∆α-helix | 86%^1^ |
| Sc^Σ^Flo11A | 122%^1^ |
| ScFlo11A^Σins^ | 83%^1^ |
| STREPII-SpFlo11A | 97%^1^; 76%^2^ |
| STREPII-KlFlo11A | 67%^2^ |
| STREPII-TdFlo11A | 96%^2^ |
| STREPII-KpFlo11A | 87%^2^ |
| STREPII-ClFlo11A | 93%^2^ |
| STREPII-MgFlo11A | 71%^2^ |

^1^Obtained by using polyclonal anti-ScFlo11A antibodies

^2^Obtained by using monoclonal anti-STREP II antibodies
